# Impact of interprofessional blended learning with low-fidelity simulation on neonatal resuscitation skills among interns: a quasi-experimental study in Tanzania

**DOI:** 10.1186/s41077-026-00451-w

**Published:** 2026-06-18

**Authors:** Ambroce Stephen, Angelina A. Joho, Vicent Bankanie

**Affiliations:** 1 Department of Clinical Nursing, School of Nursing and Public Health, University of Dodoma, Dodoma, Tanzania; 2 Department of Paediatrics, School of Nursing, Kairuki University, Dar es Salaam, Tanzania

**Keywords:** Helping Babies Breathe, Low-fidelity simulation, Blended learning, Neonatal resuscitation, Medical and nursing interns, Dar es Salaam, Tanzania

## Abstract

**Background:**

Despite the national rollout of the Helping Babies Breathe (HBB) program, neonatal mortality due to birth asphyxia remains a major challenge in Tanzania. Evidence shows that there is inadequate competence (including knowledge, skills, and attitudes) in newborn resuscitation among medical and nursing interns as they transition into practice. The traditional method of a one-day instructor-led HBB model provides little hands-on practice. Integrating low-fidelity simulation with blended learning may contribute to better transfer of learning and offer a more sustainable model for skills retention.

**Objective:**

To evaluate the efficacy of interprofessional low-fidelity simulation with a blended-learning approach among medical and nursing interns’ HBB knowledge and skills in Dar es Salaam.

**Methods:**

A controlled quasi-experimental design was used with 128 participants. Stratified random sampling by profession was used to select participants for the two groups (64 in the intervention group, 64 in the control group). The intervention group received a 2-h instructor-led online session and two days (16 h total) of hands-on low-fidelity simulation, guided by the HBB 2nd Edition curriculum (which emphasises the Golden Minute, preparation for birth, initial steps including stimulation and warming, bag-mask ventilation, and post-resuscitation care through interactive case-based scenarios and paired practice). The control group received reading materials and continued with their business-as-usual practice. Knowledge (17-item MCQ) and skills (18-item checklist) were assessed at baseline, immediately after intervention, and six weeks post-intervention for both groups. Data were analysed using IBM SPSS version 27, with an independent t-test and a linear mixed model (LMM). β coefficients represent adjusted mean differences from linear mixed models.

**Results:**

The groups were similar at baseline. The intervention group showed significant improvements compared with the control group. Knowledge score increased by β = 5.39 immediately post-intervention post-training, and by β = 3.90 in six weeks (both *p* < 0.001). Skills improved by β = 5.36 immediately and β = 3.47 at six weeks (both *p* < 0.001).

**Conclusion:**

Low-fidelity simulation with blended learning significantly improved HBB knowledge and skills among interns, with effects sustained at six weeks. This approach is practical and scalable for strengthening newborn resuscitation capacity in resource-limited settings.

**Trial registration:**

Trial registered to Pan African Trial Registry with Reg no PACTR202512785351089.

## Background

Neonatal mortality continues to be a significant global health challenge, with an estimated 17 newborn deaths per 1,000 live births reported in 2022 [1]. Sub-Saharan Africa carries the highest burden, averaging 27 deaths per 1,000 live births [2]. In Tanzania, the neonatal mortality rate (NMR) is currently estimated at 24 per 1,000 live births [3]. Recent data from Dar es Salaam, the country’s largest urban centre, shows an alarming rate of 49 neonatal deaths per 1,000 live births, the highest reported nationally [4]. The lack of sustained competence in neonatal resuscitation among intern’s accounts for a critical educational and clinical gap in Tanzania, particularly given high neonatal mortality in urban settings like Dar es Salaam. While the standard one-day HBB training improves immediate performance, skill decay occurs rapidly without reinforcement [5]. Blended learning combined with low-fidelity simulation, grounded in adult learning principles, may address this by enhancing knowledge retention, psychomotor skills, and transfer to clinical practice. However, evidence on this combined approach among Tanzanian interns remains limited.

The Helping Babies Breathe (HBB) initiative, introduced in 2010, has been adopted in more than 80 countries, including Tanzania, where national scale-up was associated with a substantial reduction in fresh stillbirths and early neonatal deaths ( [6]; Singhal et al., [7]). Despite these gains, many healthcare providers experience rapid decay in psychomotor skills, weeks after a standard one-day training [8]. Medical and nursing interns are particularly vulnerable due to limited structured supervision during rotations.

Blended learning, combining online theoretical modules with face-to-face hands-on practice, has been shown to improve knowledge retention and skills acquisition in life support training [9]. Integrating low-fidelity simulation with blended learning may offer a practical, sustainable model for resource-limited settings. Therefore, this study aims to address the specific evidence gap regarding the effectiveness of this combined approach among Tanzanian medical and nursing interns.

## Material and methods

### Study design

**Fig. 1:**
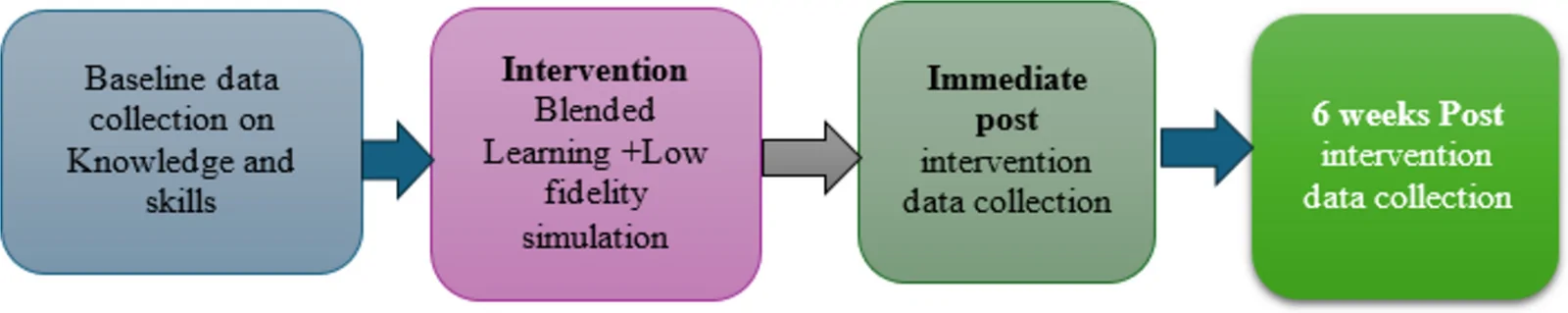
Study flow diagram

This study employed a controlled quasi-experimental design with non-equivalent groups (intervention and control). The design was selected to evaluate the causal impact of the intervention in the absence of randomization while accommodating natural variation among participants and adhering to ethical considerations. Data were collected at three points: baseline, immediate post-intervention, and six weeks post-intervention for both groups. The intervention group underwent all four study phases: baseline assessment, training intervention, immediate post-intervention assessment, and endline assessment, whereas the control group participated in all phases except the intervention [10]. Figure 1 summarizes the study phases.

This study was conducted and reported in accordance with the TREND statement for non-randomized intervention evaluations to ensure transparency and methodological rigor.

### Study setting

This study was conducted in Dar es Salaam, Tanzania’s largest city, with an estimated 6 million people, characterized by high density, informal settlements, and high neonatal morbidity and mortality driven by preterm birth complications, infections, and birth asphyxia. Significant health disparities exist, with neonatal mortality rates of 63 per 1,000 live births among the poorest versus 44 per 1,000 among the richest [11]. Muhimbili National Hospital (MNH) was selected as the intervention site due to its established newborn care facilities, high clinical workload, and routine intern rotations, making it an ideal location for HBB simulation training. In contrast, Mloganzila Hospital, a MUHAS-affiliated facility with a lower patient volume and routine intern rotation, served as the control site, providing a comparable clinical environment without exposure to the intervention.

### Study participants

The study population comprised nursing and medical doctors (MD) interns undertaking clinical practice at MNH and Mloganzila Referral Hospital in Dar es Salaam, Tanzania. These participants, who are transitioning from theoretical training to hands-on practice, were directly involved in maternal and neonatal care, including newborn resuscitation. Eligible interns were those formally enrolled in a clinical internship at either hospital during the study period, had not received HBB training within the six months preceding data collection to reduce the influence of recent exposure, and voluntarily provided informed consent after being fully informed on the study objectives, procedures, and ethical considerations and exclude interns who were not available during the study period or unable to provide commitment to the study schedule due to other obligations were excluded from the study.

### Sample size

The sample size was calculated using standard statistical parameters to ensure adequate power to detect meaningful differences, using the Rosner formula to determine the required sample size per group: *n* = 2* (Zα/2 + Zβ)2*σ​2/d2 (Rosner, [12]). With a significance level of 0.05 (Zα/2 = 1.96), power of 80% (Zβ = 0.84), and an estimated standard deviation (σ) of 1 from a prior study and a minimum effect size (Cohen’s d) of 0.5, the formula yielded an initial sample size of approximately 53 participants per group. After adjusting for a 10% non-response rate to account for potential attrition, the final minimum sample size was set at 64 participants per group, resulting in a total of 128 participants, with equal allocation to the intervention and control arms (1:1 ratio).

### Recruitment of participants

#### Selection of study participants

A stratified random sampling technique by profession was employed to ensure balanced representation of both medical Doctors (MD) and nursing interns. Participants were first grouped according to their profession, and random selection within each group was conducted using the RAND function in Microsoft Excel 365. Following selection, participants were assigned to study groups based on their internship hospital placement, whereby interns from Muhimbili National Hospital were allocated to the intervention group, while those from Mloganzila Hospital were allocated to the control group, in line with the non-randomized quasi-experimental study design. This method minimized selection bias and ensured that the sample adequately represented the diversity of interns across the two hospitals.

**Figure:**
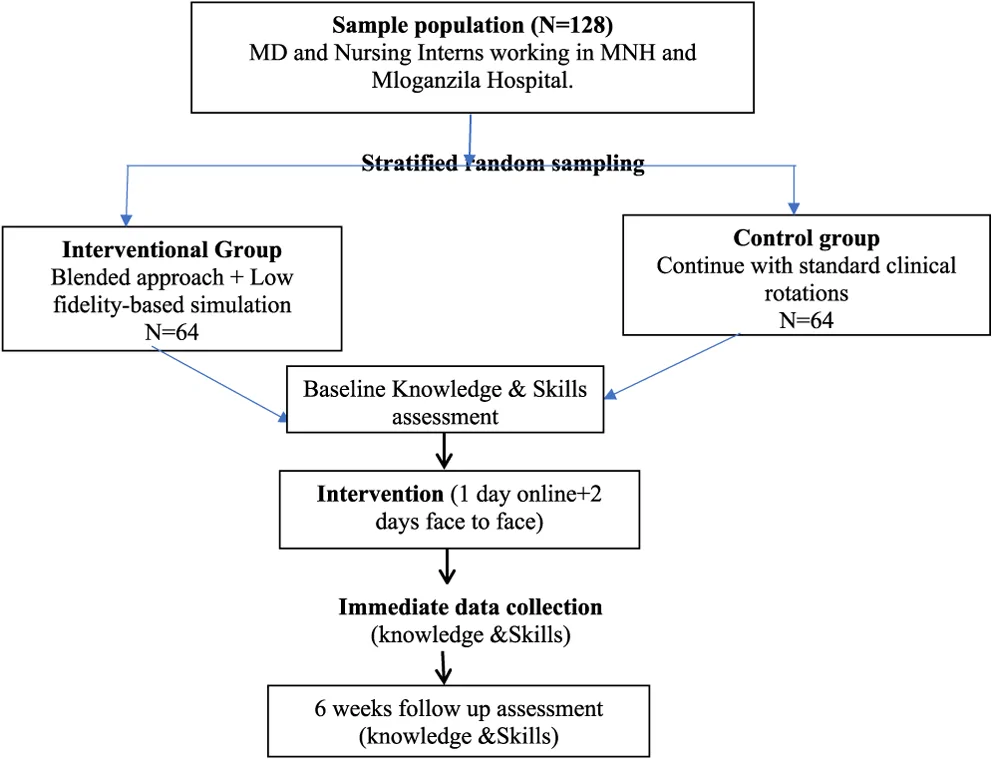

### Study flow diagram

#### Recruitment and training of HBB expert and research assistant

HBB facilitators were recruited based on national certification as Helping Babies Breathe trainers accredited by the Ministry of Health, Tanzania. Eligibility criteria included prior experience delivering at least two HBB training sessions in clinical or educational settings. To ensure standardization and high instructional quality, selected facilitators were certified by the Ministry of Health as national HBB facilitators, and they participated in a one-day refresher orientation led by the principal investigator. The orientation covered a review of the HBB 2nd edition curriculum, simulation-based teaching methods, and study-specific procedures. Due to the nature of the educational intervention, blinding of participants and HBB facilitators was not feasible, as both were aware of the training approach being implemented. Interns in the intervention group received low-fidelity simulation training integrated with a blended learning approach, while those in the control continue with their clinical rotation. However, to minimize assessment bias, outcome assessors were not involved in the delivery of the intervention and used standardized assessment tools and checklists during data collection. In addition, participants were assessed under similar conditions across both study sites to ensure consistency and objectivity in outcome measurement.

### Description of the intervention

#### Pre-Intervention phase

During this phase, HBB training materials were adapted into a blended format by integrating online and face-to-face components, updating resources, and designing practical simulation scenarios. Awareness was raised through meetings with hospital administrators, and experts ensured alignment with institutional policies and ethical approval. An implementation team of certified HBB facilitators and four trained research assistants was established and oriented on the protocol, tools, and training delivery to ensure methodological rigor and logistical readiness. Also, eligible MD and nurse interns were recruited from MNH (intervention) and Mloganzila Referral Hospital (control), with baseline assessments conducted using a 17-item knowledge questionnaire and an 18-item skills checklist. For the intervention group, a Google Meet platform was created to host training modules and guidelines, enabling self-paced learning before the simulation sessions**.**

#### The intervention

The intervention employed a blended learning approach integrating online instruction with face-to-face, low-fidelity simulation-based training to enhance participants' knowledge and practical skills in neonatal resuscitation, as outlined in the HBB curriculum.

The intervention was guided by Kolb’s Experiential Learning Theory, which structures learning through four stages. Abstract conceptualisation was addressed via the 2-h instructor-led online session covering HBB theoretical principles. Concrete experience occurred over two days of hands-on, low-fidelity simulation in which interns practised resuscitation procedures on NeoNatalie mannequins using case-based scenarios. Reflective observation was facilitated through structured debriefing with certified HBB trainers and peer-to-peer feedback after each simulation round. Active experimentation enabled learners to immediately apply corrected techniques in repeated practice cycles, alternating roles and refining performance. The 18-h blended format (2 h online + 16 h in-person) deliberately allowed multiple complete cycles of the Kolb loop, moving interns continuously between thinking and doing, reflecting and acting—this theory-driven design aimed to produce durable, transferable competence in neonatal resuscitation.

#### Online component

The online portion consisted of a two-hour, instructor-led session conducted via Google Meet, focusing on the theoretical aspects of HBB. Google Meet was selected for its accessibility across Tanzania and its features that support interactive learning, including live discussion, screen sharing for instructional materials, and real-time Q&A. Although the platform does not offer direct offline access, all sessions were recorded and shared with participants experiencing intermittent internet connectivity, thereby improving accessibility for those in low-resource settings.

#### In-person simulation component

The face-to-face simulation training was conducted in a designated conference room within the Pediatric Department at Muhimbili National Hospital. The room was equipped with NeoNatalie low-fidelity simulators and other Helping Babies Breathe (HBB) training materials to facilitate hands-on practice, small-group learning, and simulation-based activities.

The face-to-face component took place over two consecutive days (8 h per day), adhering to the American Academy of Paediatrics’ (AAP) HBB 2nd Edition curriculum. The training component was delivered by trained Helping Babies Breathe (HBB) facilitators who were certified by the Ministry of Health, Tanzania, as national HBB trainers. The sessions were conducted by a team of three facilitators consisting of one HBB expert, one medical officer facilitator, and one nursing officer facilitator under the supervision of the principal investigator to ensure adherence to the American Academy of Pediatrics (AAP) HBB 2nd Edition curriculum. Participants were organized into small groups of approximately 5–8 interns during the session, allowing close supervision and active participation during simulation practice.

Each group rotated through stations for demonstration, hands-on practice using NeoNatalie simulators, peer-to-peer feedback, and guided skill reinforcement, ensuring adequate practice time and individualized feedback for all participants.

The total contact time for the intervention group was approximately 18 h (2 h online + 16 h in-person), exceeding the AAP’s standard one-day (8-h) HBB course [13]. This extended duration was purposefully designed to reinforce both knowledge acquisition and skill retention through repeated practice and immediate feedback.

Extending the simulation into two 8-h sessions was both evidence-based and contextually justified to strengthen psychomotor competence and allow adequate time for repetitive practice and reflective debriefing. McGaghie et al. [14] demonstrated that shorter, distributed simulation sessions enhance procedural skill retention more effectively than single, extended sessions by minimising cognitive overload and supporting spaced learning. Similarly, Campain et al. [15] reported that capping simulation sessions at approximately 4–5 h per day optimises learner engagement and improves psychomotor performance in surgical and resuscitation training contexts.

To enhance compliance with the intervention, several supportive strategies were implemented. Training sessions were scheduled in collaboration with internship coordinators and clinical supervisors to align with duty rosters and minimise disruption of clinical responsibilities. Participants were given protected time for training, attendance was formally recorded, and refreshments were provided during the training. No financial incentives were provided.

##### Scheduling and feasibility

Training sessions were scheduled in collaboration with hospital internship coordinators and department supervisors to respect clinical rosters. Sessions were organised in blocks and aligned with duty schedules to maintain clinical coverage. Prior approvals were obtained from hospital supervisors, and attendance logs were kept.

### Control group

The control group received reading material (brochures) and continued their routine internship rotations as per their respective interns' rotation schedule. This approach reflects the typical internship experience in Tanzania, serving as a pragmatic comparator to the structured intervention. This distinction allowed for a clear comparison between structured blended training and routine experiential learning, highlighting the added value of the intervention.

### Post-intervention phase

The post-intervention phase focused on evaluating the impact of the training by assessing changes in participants' knowledge and skills at two key time points. The first assessment involved immediate post-intervention data collection for both the intervention and control groups to measure the short-term efficacy of the respective training approaches.

Participants completed the same 17-item multiple-choice questionnaire (MCQ) to evaluate improvements in their theoretical knowledge of the HBB program. Additionally, their practical neonatal resuscitation skills were reassessed using an 18-item skills-based checklist. For the intervention group, this evaluation measured the effectiveness of the blended learning strategy combined with low-fidelity simulation training. In contrast, the control group continues with their normal clinical rotation.

This immediate post-intervention assessment helped determine whether the educational objectives were achieved, particularly in terms of how effectively the simulation-based training conveyed key concepts and essential practices in neonatal resuscitation [16].

At six weeks post-intervention, data collection was conducted to assess the retention of knowledge and skills in neonatal resuscitation among study participants. The same 17-item multiple-choice questionnaire (MCQ) and 18-item skills-based checklist previously used during baseline and immediate post-intervention assessments were re-administered.

This phase aimed to evaluate both the improvement and sustainability of participants’ knowledge and practical skills over time. The findings provided valuable insight into the long-term effectiveness of the blended learning approach combined with low-fidelity simulation, particularly in maintaining critical competencies in HBB among intern doctors and nurses [17].

By comparing the immediate and six-week post-intervention data between the two groups, the study generated robust evidence on the effectiveness of blended learning combined with low-fidelity simulation in enhancing the HBB competencies of MD and Nurse Interns in Tanzania (Table 1).

**Table 1:** Summary of the intervention

Phase		Activity
Preparation phase		• Adapted training materials were prepared to align with the HBB curriculum
		• Informational brochures summarizing key concepts were produced for participants and stakeholders
		• A meeting was held with administrative authorities and HBB experts to provide a detailed explanation of the research, address ethical considerations, and secure institutional support
		• The research team, comprising trainers, facilitators, and data collectors, was assembled and trained in study protocols and procedures
Pre-Intervention Phase		• The sampling procedure was conducted
		• Baseline data collection was completed for both groups
		• An online platform for sharing educational materials was created
Intervention	Intervention Group	• Pre-session online material was conducted (1 day)
		• Simulation using low fidelity (2 days)
	Control group	• Only reading material(brochures)
Post intervention		• Immediate post data collection (knowledge skills) to both groups
		• Six weeks post-intervention data collection (knowledge skills) to both groups (Endline data collection)

### Intervention package

The intervention package was adapted from the American Academy of Pediatrics’ (AAP) HBB curriculum and delivered through a blended learning approach. A train-the-trainer model was employed to ensure consistency, with expert trainers preparing facilitators to deliver standardized sessions. Low-fidelity simulators were used, with one simulator assigned to each pair of trainees to maximize hands-on practice. Training followed a cascade model, engaging master trainers, facilitators, and learners. Participants alternated between performing neonatal resuscitation procedures on simulators and providing peer feedback, thereby reinforcing both technical skills and teaching competencies [18].

### Strategies to improve adherence to the intervention

Before the intervention, clinical supervisors and ward in-charges were briefed on study procedures, and hospital administrators issued internal memos authorizing interns’ release from routine duties to ensure protected time for training and assessments. To enhance participation, personalized mobile text-message reminders were sent 24 h before each session, and again 1 h before, with attendance registers maintained throughout. Absentees were followed up via phone, provided with recorded online sessions, and offered make-up opportunities where feasible. The training emphasized interactive, case-based scenarios and hands-on simulations aligned with clinical practice. These features, grounded in adult learning theory, were designed to enhance engagement, motivation, and meaningful participation.

### Data collection methods and procedures

Knowledge assessed using a 17-item multiple-choice questionnaire adapted from the HBB 2nd Edition Facilitator Guide, which remains the most current publicly available version as of 2022. This tool covering routine newborn care, initial resuscitation, positive-pressure ventilation, and post-resuscitation management was validated through expert review and pilot testing, with ≥ 14 (82%) marks indicating adequate knowledge (based on benchmarks in prior HBB evaluations). Practical skills were evaluated using an 18-item observational checklist aligned with the HBB 2nd Edition Action Plan and OSCE guidelines, assessing steps such as preparation, stimulation, airway management, and bag-mask ventilation; a score of ≥ 15 (83%) denoted adequate practice, in line with previous Tanzanian validation studies. Trained facilitators conducted these assessments under standardized simulation conditions using NeoNatalie low-fidelity mannequins to ensure objectivity and comparability.

### Outcome measurement

#### Knowledge of HBB

The knowledge assessment comprised 17 multiple-choice questions (MCQs), each awarded one mark, for a total possible score of 17. A score of 14 or higher (82%) was used as the threshold for adequate knowledge, as was used in a previous study [19].

#### Skills in HBB

For the skills assessment, an 18-item observation checklist was used, adapted from the Helping Babies Breathe (HBB) Provider Action Plan developed by the American Academy of Pediatrics [13]. A score of 15 or more out of 18 (≥ 83%) was considered indicative of competence in neonatal resuscitation skills. This threshold is supported by Perlman et al. [20], who applied the same benchmark in their evaluation of HBB implementation in Tanzania and found it to have strong predictive validity for post-training clinical performance. The 83% cut-off corresponds to correctly performing at least five out of six critical steps, thereby ensuring retention of key life-saving actions such as initiating ventilation within the Golden Minute. This performance benchmark has also been consistently adopted in other HBB studies conducted in low-resource settings, further validating its relevance for assessing practical competence in neonatal care [21].

### Validity and reliability

The 17-item knowledge questionnaire and 18-item skills checklist were adopted from the internationally and nationally standardized American Academy of Pediatrics’ HBB 2nd edition curriculum [13], with the addition of a demographic section. The standardized instruments were reviewed and adapted to suit the local Tanzanian context, ensuring they accurately measured the intended constructs of knowledge and skills in Helping Babies Breathe. The reliability of the adopted tools from the internationally and nationally standardized American Academy of Pediatrics’ HBB 2nd edition curriculum was ensured through a pilot study conducted at Muhimbili National Hospital with 14 intern doctors and nurses (approximately 10% of the target sample). The pilot assessed the clarity, cultural relevance, and applicability of the tools in the local context.

A study adapting HBB into a virtual curriculum reported excellent internal reliability with Cronbach's alpha values of 0.89 in confidence surveys [22]. Inter-rater calibration was also performed for the skills checklist to improve scoring consistency during the main study. These findings increased confidence in the use of the instruments for the main study.

### Data analysis

Data were analysed using IBM SPSS Statistics version 27. Normality was assessed with Shapiro–Wilk and Kolmogorov–Smirnov tests, supported by visual inspection. Intervention effects on knowledge and skills over time (baseline, immediate post, and six-week follow-up) were examined using an independent t-test and Linear Mixed Model (LMM), with fixed effects for time, group, and their interaction. Participant ID was modelled as a random intercept, with statistical significance set at *p* < 0.05 and results reported with 95% confidence intervals.

## Results

### Social-demographic characteristics of participants

A total of 128 participants (64 in the control and 64 in the intervention) were involved. Participants in the intervention and control groups were demographically comparable at baseline. Table 2 summarizes the baseline demographic characteristics of participants in both the intervention and control groups.

**Table 2:** Distribution of study participants by social demographic characteristics

Variable	Intervention group (*n* = 64)	Control group (*n* = 64)	*p*-value
Mean age (years) ± SD (range)	26.10 ± 1.71 (23–32)	25.78 ± 1.88 (22–31)	0.215
Age (years)			
23- 26	35 (54.6%)	41 (64.1%)	0.129
27–32	29 (45.4%)	23 (35.9%)	
Sex			
Male	32 (50%)	30 (46.9%)	0.724
Female	32 (50%)	34 (53.1%)	
Intern			
MD	32 (50%)	32 (50%)	1.000
Nurse	32 (50%)	32 (50%)	
Entry qualification			
Direct	60 (93.8%)	60 (93.8%)	1.000
Equivalent	4 (6.3%)	4 (6.3%)	
University			
Public	28 (43.8%)	27 (42.2%)	0.85
Private	36 (56.2%)	37 (57.8%)	
Prior HBB Training			
Yes	40 (62.5%)	35 (54.7%)	0.370
No	24 (37.5%)	29 (45.3%)	
Exposed to HBB from the Media			
Yes	16 (25%)	21 (32.8%)	0.330
No	48 (75%)	43 (67.2%)	
Completed Labor Ward Rotation			
Yes	34 (53.1%)	27 (42.2%)	0.215
No	30 (46.9%)	37 (57.8%)	

#### Baseline knowledge and skills

At baseline, there was no significant difference in knowledge scores between the intervention (10.19 ± 1.99) and control (9.98 ± 2.31) groups, t (126) = 0.52, *p* = 0.607. The effect size was negligible (Cohen's d = 0.09), confirming group comparability before the intervention (Table 3). Similarly, the baseline skills scores of the intervention group and the control group did not differ significantly.

**Table 3:** Baseline knowledge and skills on helping babies breathe between medical doctors and nurses interns in control and intervention groups

Variable	Control (M ± SD)	Intervention (M ± SD)	Mean Difference	Std. Error	95% CI		t-value	p-value	Cohen's d
					**Lower**	**Upper**			
Knowledge Score	9.98 ± 2.31	10.19 ± 1.99	0.20	0.38	−0.56	0.95	0.52	0.607	0.09
Skills Score	8.95 ± 2.45	9.06 ± 2.45	0.11	0.47	−0.03	1.84	1.92	0.057	0.34

### The effect of interprofessional blended learning with low-fidelity simulation on the knowledge of intern medical doctors and nurses regarding neonatal resuscitation

Table 4 below shows that at baseline, knowledge scores were comparable between groups. Immediately after training, the intervention group demonstrated significantly higher knowledge scores than the control group (intervention M = 16.00 ± 1.01 vs control M = 10.42 ± 1.98; mean difference = 5.59, 95% CI = 5.04 to 6.13, t = 20.09, *p* < 0.001, Cohen’s d = 3.55). At endline, the intervention group retained substantially higher knowledge (intervention M = 14.06 ± 1.10 vs control M = 9.96 ± 2.13; mean difference = 4.10, 95% CI = 3.51 to 4.69, t = 13.71, *p* < 0.001, Cohen’s d = 2.42).

**Table 4:** Overall mean score changes and difference in HBB knowledge between baseline, immediate, and end line among intern nurses and doctors

Time Point	Control (M ± SD)	Intervention (M ± SD)	Mean Diff	Std. Error	95% CI		t-value	*p*-value	Cohen's d
					**Lower**	**Upper**			
Baseline	9.98 ± 2.31	10.19 ± 1.99	0.2	0.38	−0.56	0.95	0.52	0.607	0.09
Immediate Post Intervention	10.42 ± 1.98	16.00 ± 1.01	5.59	0.28	5.04	6.14	20.09	< 0.001	3.55
Endline	9.96 ± 2.13	14.06 ± 1.10	4.1	0.3	3.51	4.69	13.71	< 0.001	2.42

### Linear mixed model estimates for knowledge scores

Table 5 below shows that the intervention was effective in the immediate period and remained significant at the endline. At baseline, the intervention and control groups did not differ significantly in knowledge score (β = 0.20, CI −0.56–0.95, *p* = 0.607). Immediately after the intervention, the knowledge score showed no significant change compared to baseline (β = 0.03, *p* = 0.918, CI −0.66–0.59). At the endline, there was a slight increase in knowledge compared to baseline; however, it was not statistically significant (β = 0.42, *p* = 0.187, CI −0.21–1.05). Interaction effects in the immediate post-intervention period show a statistically significant improvement in the knowledge score of HBB compared to the control group (β = 5.39, *p* < 0.001). At the endline assessment, the intervention group still maintained a significant improvement in knowledge scores compared to the control group. However, the effect size was smaller than at the immediate time point (CI: 3.02–4.79).

**Table 5:** Linear mixed model estimates for knowledge scores

Effect	Estimate (β)	Std. Error	t-value	*p*-value	95% CI	
					**Lower**	**Upper**
Intercept	9.99	0.27	36.9	< 0.001	9.45	10.52
Group						
Intervention	0.20	0.38	0.15	0.607	−0.56	0.95
Control	Reff					
Time						
Immediate	0.03	0.32	0.10	0.918	−0.66	0.59
Endline	0.42	0.32	1.32	0.187	−0.21	1.05
Baseline	Reff					
Interaction: Time × Group						
Immediate × Intervention	5.39	0.45	11.96	< 0.001	4.50	6.28
Endline × Intervention	3.90	0.45	8.98	< 0.001	3.02	4.79
Baseline × Intervention	Reff					

### Effect of the intervention on HBB skills

Table 6 shows that at baseline, the control and intervention groups had similar skill levels (8.95 ± 2.45 and 9.06 ± 2.45, respectively), with a mean difference = 0.11, *p* = 0.800, and Cohen’s d = 0.05. Immediately post-intervention, there was a great and statistically significant improvement in skills with a mean difference = 6.78, *p* < 0.001, Cohen’s d = 2.98. During follow-up, the skills score was retained significantly in the intervention group compared to the control group, with a mean difference = 5.15, *p* < 0.001, Cohen’s d = 2.28.

**Table 6:** Comparison of skills scores between control and intervention groups at baseline, immediate post-training, and endline

Time Point	Control (M ± SD)	Intervention (M ± SD)	Mean Difference	Std. Error	95% CI of Difference		t-value	*p*-value	Cohen's d
					**Lower**	**Upper**			
Baseline	8.95 ± 2.45	9.06 ± 2.45	0.11	0.43	−0.745	0.97	0.25	0.800	0.05
Immediate Post	9.00 ± 2.45	15.78 ± 2.08	6.78	0.40	5.99	7.58	16.88	< 0.001	2.98
Endline	8.65 ± 2.40	13.80 ± 2.10	5.15	0.40	4.36	5.94	12.92	< 0.001	2.28

Using a linear mixed model, demonstrated that skills in the intervention improved significantly. At baseline, the intervention and control groups' skills scores were equivalent (β = 0.96, CI −0.02–1.84, *p* = 0.570). Immediately after the intervention, the skills score improved significantly compared to the control group (β = 5.06, *p* < 0.001, 95% CI = 3.87–6.24). During follow-up, the intervention group maintained a significant improvement in skills scores compared to the control group (β = 3.87, *p* < 0.001, 95% CI = 2.69–5.05) (Table 7).

**Table 7:** Linear mixed model estimates for skills scores (reference categories: group = control, time = baseline)

Effect	Estimate (β)	Std. Error	t	*p*-value	95% CI	
					**Lower**	**Upper**
Intercept	8.95	0.33	26.8	< 0.001	8.29	9.61
Group						
Intervention	0.96	0.47	1.92	0.570	−0.02	1.84
Control	Ref					
Time × Group						
Immediate ×						
Intervention	5.06	0.6	8.42	**< 0.001**	3.87	6.24
Control	Ref					
Endline ×						
Intervention	3.87	0.59	6.4	**< 0.001**	2.69	5.05
Baseline	Ref					

## Discussion

This study evaluated the efficacy of combining interprofessional blended learning with low-fidelity simulation on the Helping Babies Breathe (HBB) knowledge and skills of Tanzanian medical doctors and nurse interns. Compared to the control group, the interventional group demonstrated a significant and sustained increase in both knowledge and skills at six weeks, with a large effect size.

### Knowledge outcomes

Immediately after training, the intervention group scored significantly higher in knowledge compared to the control (16.00 ± 1.01 vs. 10.42 ± 1.98, d = 3.55, *p* < 0.001) and remained significantly higher at endline (14.06 ± 1.10 vs. 9.96 ± 2.13, d = 2.42, *p* < 0.001). These findings reflect the effectiveness of the blended approach in improving knowledge. The combined online pre-learning with hands-on simulation allowed participants to review materials independently before practicing. This design aligns with Kolb's experiential learning theory, which emphasizes conceptual understanding through iterative cycles of conceptualization and practice [23].

This further highlights the value of this approach in boosting learning and helping interns retain critical knowledge, supplementing the knowledge gap in their prior training.

Consistent with this study, a recent systematic review reinforces that simulation, even at low fidelity, is a powerful tool for improving knowledge [24]. Similar findings have also been repeatedly reported elsewhere [25, 26]. Contrary to this study, A study conducted in India by Kc et al., [27] found that, while initial knowledge gains were evident post-HBB training, these gains diminished substantially over time in the absence of reinforcement or structured follow-up. However, the integration of a blended learning component and a structured follow-up in this study may have provided additional cognitive support and learner autonomy factors that likely contributed to more sustained knowledge acquisition over time, unlike in other studies [18].

### Skills outcomes

In this study, improvement in participants’ skills mirrored knowledge gains. The intervention group revealed marked improvement immediately post-training (15.78 ± 2.08 vs. 9.00 ± 2.45, d = 2.98, *p* < 0.001) and maintained superiority at six weeks (13.80 ± 2.10 vs. 8.65 ± 2.40, d = 2.28, *p* < 0.001). The repeated hands-on practices with real-time feedback strengthened procedural memory and reduced anxiety. This enabled meaningful skill application.

Consistently, a study by Ding et al. [28] demonstrated that structured neonatal resuscitation simulation training produced significant gains in skills. This highlights the reliability of simulation-based training as a proven way to build and sustain critical lifesaving skills, even in low-resource environments [28, 29]. Notably, the sustained effects observed at six weeks contrast with earlier reports of rapid skill decay [30], and support the feasibility of low-dose, high-frequency simulation in resource-limited settings [8].

An important outcome was the reduced competency gap between nurses and medical interns after training. This mirrors findings from educational interventions in low-resource neonatal care where interprofessional simulation promotes shared mental models, equal participation, and role clarity [31]. In Tanzania, similar patterns have been reported where simulation reduces hierarchical learning disparities and improves collaborative practice during delivery emergencies [8]. Equalizing skills among cadres is vital in labor wards, where nurses and interns frequently work interchangeably during neonatal emergencies.

## Conclusion and recommendation

This study suggests that low-fidelity simulation integrated with blended learning can significantly improve and sustain HBB competence among interns in resource-limited settings.

Institutions should consider incorporating structured simulation and digital pre-learning into intern training programs, supported by trained facilitators. Policymakers and professional bodies are encouraged to evaluate and potentially adapt national HBB guidelines to include reinforced, distributed training models**.** Future research should examine long-term retention (> 6 months), real-clinical performance (rather than simulation), cost-effectiveness, scalability in rural settings, and comparative trials with matched time-on-task or alternative active controls to disentangle the unique contributions of blended learning and low-fidelity simulation formats.

### Study limitations

This study faced several limitations. The quasi-experimental design lacked randomization, which may have introduced uncontrolled confounding factors. The control group received no structured training, and the study involved only interns in Dar es Salaam, limiting generalizability to other settings. The short follow-up period prevented assessment of long-term knowledge and skill retention. Moreover, skills were assessed through simulation rather than real-life practice. Despite these limitations, the study provides meaningful insights into the potential of blended low-fidelity simulation to strengthen HBB competencies among Tanzanian interns.

## Data Availability

No datasets were generated or analysed during the current study.

## References

[1] Hug L, Liu Y, Nie W, Sharrow D, You D, Cao B, Ma Fat D, Ho J, Retno Mahanani W, Strong K, Wang World Bank Group Emi Suzuki H, Butler D, Dorion C, Gerland P, Hertog S, Kamiya Y, Kantorova V, Kyaw Lay K, Lattes P, Guillot M. Levels & trends in child mortality. Report 2023. https://data.unicef.org/resources/levels-and-trends-in-child-mortality-2023/.

[2] Ahmed KY, Thapa S, Hassen TA, Tegegne TK, Dadi AF, Odo DB, et al. Population modifiable risk factors associated with neonatal mortality in 35 sub-Saharan Africa countries: analysis of data from demographic and health surveys. EclinicalMedicine. 2024;73:1–12. 10.1016/j.eclinm.2024.102682. PMC11245992 39007064

[3] Shabani J, Salim N, Bohne C, Day LT, Kumalija C, Makuwani AM, et al. Neonatal indicator data in Tanzania District Health Information System: evaluation of availability and quality of selected newborn indicators, 2015-2022. BMC Pediatr. 2025;23:658. 10.1186/s12887-025-05417-x. 39849367 PMC11755859

[4] Kagoye S, Minja J, Ricardo L, Shabani J, Bajaria S, Msuya S, et al. High child mortality and interventions coverage in the city of Dar es Salaam, Tanzania: are the poorest paying an urban penalty? J Urban Health. 2024;101(1):92–106. 10.1007/s11524-023-00813-z. 38216824 PMC11602923

[5] Mayer MM, Xhinti N, Mashao L, Mlisana Z, Bobotyana L, Lowman C, Patterson J, Perlman JM, Velaphi S. Effect of Training Healthcare Providers in Helping Babies Breathe Program on Neonatal Mortality Rates. Front Pediatr. 2022;10. 10.3389/fped.2022.872694. PMC9158330 35664883

[6] Msemo G, Massawe A, Mmbando D, Rusibamayila N, Manji K, Kidanto HL, et al. Newborn mortality and fresh stillbirth rates in Tanzania after Helping Babies Breathe training. Pediatrics. 2013;131(2):e353–60. 23339223 10.1542/peds.2012-1795

[7] Singhal, Nalini, Jocelyn Lockyer, Herta Fidler, William Keenan, George Little, Sherri Bucher, Maqbool Qadir, and Susan Niermeyer. 2012. “Helping Babies Breathe: Global Neonatal Resuscitation Program Development and Formative Educational Evaluation.” Resuscitation 83 (1): 90–96. 10.1016/j.resuscitation.2011.07.010 21763669

[8] Kalabamu FS, Daudi V, Moshiro R, Kamala B, Mdoe P, Bishanga D, et al. Neonatal resuscitation skills acquisition among healthcare providers after Helping Babies Breathe simulation training using improved tools across two regions in Tanzania. Adv Simul. 2025;10(1):1–10. 10.1186/s41077-025-00338-2. PMC12362981 40025598

[9] Abuejheisheh AJ, Alshraideh JA, Amro N, Bani Hani S, Darawad MW. Effectiveness of blended learning basic life support module on knowledge and skills: a systematic review of randomized controlled trials. Heliyon. 2023;9(11):e21680. 10.1016/j.heliyon.2023.e21680. 38027704 PMC10661193

[10] Joho AA, Kibusi SM, Mwampagatwa I. Predictors of Helping Babies Breathe knowledge and skills among nurses in primary health settings in Dodoma region. Tanzania. 2020;9:1–7. 10.1186/s12884-020-2782-9 PMC7068969 32164561

[11] United Nations Inter-agency Group for Child Mortality Estimation (UN IGME). (2023). *Levels and trends in child mortality: Report 2023*. UNICEF. https://data.unicef.org/resources/levels-and-trends-in-child-mortality-2023

[12] Rosner, Bernard, Robert J Glynn, and Mei-ling T Lee. 2006. “The Wilcoxon Signed Rank Test for Paired Comparisons of Clustered Data,” no. March: 185–92. https://academic.oup.com/biometrics/article-abstract/62/1/185/7306230 10.1111/j.1541-0420.2005.00389.x 16542245

[13] American Academy of Pediatrics. *Helping babies breathe*. 2nd ed. American Academy of Pediatrics. 2022.

[14] McGaghie WC, Issenberg SB, Petrusa ER, Scalese RJ. A critical review of simulation-based medical education research: 2003-2009. Med Educ. 2010;44(1):50–63. 10.1111/j.1365-2923.2009.03547.x. 20078756

[15] Campain NJ, Kailavasan M, Chalwe M, Gobeze AA, Teferi G, Lane R, et al. An Evaluation of the Role of Simulation Training for Teaching Surgical Skills in Sub-Saharan Africa. World J Surg. 2018;42(4):923–9. 10.1007/s00268-017-4261-7. 29026963 PMC5843670

[16] Shin S, Park JH, Kim JH. Effectiveness of patient simulation in nursing education: meta-analysis. Nurse Educ Today. 2015;35(1):176–82. 10.1016/j.nedt.2014.09.009. 25459172

[17] Vossius C, Lotto E, Lyanga S, Mduma E, Msemo G, Perlman J, et al. Cost-effectiveness of the “Helping Babies Breathe” program in a Missionary Hospital in rural Tanzania. PLoS ONE. 2014;9(7):7–12. 10.1371/journal.pone.0102080. PMC4090230 25006802

[18] Goudar SS, Somannavar MS, Clark R, Lockyer JM, Revankar AP, Fidler HM, Sloan NL, Niermeyer S, Keenan WJ, Singhal N. Stillbirth and newborn mortality in india after helping babies breathe training. Pediatrics. 2013;131(2). 10.1542/peds.2012-2112. 23339215

[19] Chaulagain DR, Malqvist M, Wrammert J, Gurung R, Brunell O, Basnet O, et al. Service readiness and availability of perinatal care in public hospitals - a multi-centric baseline study in Nepal. BMC Pregnancy Childbirth. 2022;22(1):1–10. 10.1186/s12884-022-05121-z. 36380317 PMC9667676

[20] Perlman J, Msemo G, Ersdal H, Ringia P. Designing and implementing the Helping Babies Breathe Program in Tanzania. J Pediatr Intensive Care. 2016;06(01):028–38. 10.1055/s-0036-1584674. PMC6260262 31073423

[21] Bang A, Patel A, Bellad R, Gisore P, Goudar SS, Esamai F, Liechty EA, Meleth S, Goco N, Niermeyer S, Keenan W, Kamath-rayne BD, Little GA, Clarke SB, Flanagan VA, Bucher S, Jain M, Mujawar N, Jain V, Hibberd PL. Helping Babies Breathe ( HBB ) training : What happens to knowledge and skills over time ? BMC Pregnancy Childbirth. 2016. 10.1186/s12884-016-1141-3. PMC5120476 27875999

[22] Sobelman C, Richard K, McQuilkin P, Fahey N. Adapting Helping Babies Breathe into a Virtual Curriculum: Methods, Results, and Lessons Learned. Glob Pediatr Health. 2021;8. 10.1177/2333794X211019698. PMC8172947 34104697

[23] Johnson C, Shen E, Winn K, Digiacobbe G, Akinola M. Neonatal resuscitation: a blended learning curriculum for medical and physician assistant students. MedEdPORTAL. 2020;16:10921. 10.15766/mep_2374-8265.10921. 32704535 PMC7373351

[24] Alharbi A, Nurfianti A, Mullen RF, McClure JD, Miller WH. The effectiveness of simulation-based learning (SBL) on students’ knowledge and skills in nursing programs: a systematic review. BMC Med Educ. 2024;24(1). 10.1186/s12909-024-06080-z. PMC11459713 39375684

[25] Chan NHM, Merali HS, Mistry N, Kealey R, Campbell DM, Morris SK, et al. Development of a novel mobile application, HBB Prompt, with human factors and user-centred design for Helping Babies Breathe skills retention in Uganda. BMC Med Inform Decis Mak. 2021;21(1):1–15. 10.1186/s12911-021-01406-z. 33541340 PMC7863544

[26] Gangji RR, Malasa L, Koya GD, Khaki A, Rutachunzibwa F, Fataki M, Kalabamu FS, Johnston EM, Mwaikambo E. Peer-Led Neonatal Resuscitation Training: Experience of Kairuki University. J Med Educ Curric Dev. 2025;12. 10.1177/23821205251358037. PMC12290356 40717904

[27] Kc A, Wrammert J, Nelin V, Clark RB, Ewald U, Peterson S. Evaluation of Helping Babies Breathe Quality Improvement Cycle ( HBB-QIC ) on retention of neonatal resuscitation skills six months after training in Nepal. 2017;1–9. 10.1186/s12887-017-0853-5. PMC5387236 28399847

[28] Ding X, Wang L, Msellem MI, Hu Y, Qiu J, Liu S, et al. Evaluation of a neonatal resuscitation training programme for healthcare professionals in Zanzibar, Tanzania: a pre-post intervention study. Front Pediatr. 2021;9:1–8. 10.3389/fped.2021.693583. PMC8273261 34262890

[29] Al-Wassia H, Bamehriz M, Atta G, Saltah H, Arab A, Boker A. Effect of training using high-versus low-fidelity simulator mannequins on neonatal intubation skills of pediatric residents: a randomized controlled trial. BMC Med Educ. 2022;22(1):1–7. 10.1186/s12909-022-03572-8. 35752776 PMC9233370

[30] Bellad RM, Bang A, Carlo WA, McClure EM, Meleth S, Goco N, et al. A pre-post study of a multi-country scale up of resuscitation training of facility birth attendants: Does Helping Babies Breathe training save lives? BMC Pregnancy Childbirth. 2016;16(1):1–10. 10.1186/s12884-016-0997-6. 27527831 PMC5477802

[31] Elgohary M, Palazzo FS, Breckwoldt J, Cheng A, Pellegrino J, Schnaubelt S, et al. Blended learning for accredited life support courses – A systematic review. Resuscitation Plus. 2022;10:100240. 10.1016/j.resplu.2022.100240. 35592876 PMC9112020

